# Isolation of novel coregulatory protein networks associated with DNA-bound estrogen receptor alpha

**DOI:** 10.1186/1471-2199-9-97

**Published:** 2008-10-30

**Authors:** Jennifer R Schultz-Norton, Yvonne S Ziegler, Varsha S Likhite, John R Yates, Ann M Nardulli

**Affiliations:** 1 Department of Molecular and Integrative Physiology, University of Illinois at Urbana-Champaign, Urbana, IL 61801, USA; 2 Department of Biochemistry, University of Illinois at Urbana-Champaign, Urbana, IL 61801, USA; 3 Department of Cell Biology, The Scripps Institute, LaJolla, California 92037, USA

## Abstract

**Background:**

DNA-bound transcription factors recruit an array of coregulatory proteins that influence gene expression. We previously demonstrated that DNA functions as an allosteric modulator of estrogen receptor α (ERα) conformation, alters the recruitment of regulatory proteins, and influences estrogen-responsive gene expression and reasoned that it would be useful to develop a method of isolating proteins associated with the DNA-bound ERα using full-length receptor and endogenously-expressed nuclear proteins.

**Results:**

We have developed a novel approach to isolate large complexes of proteins associated with the DNA-bound ERα. Purified ERα and HeLa nuclear extracts were combined with oligos containing ERα binding sites and fractionated on agarose gels. The protein-DNA complexes were isolated and mass spectrometry analysis was used to identify proteins associated with the DNA-bound receptor. Rather than simply identifying individual proteins that interact with ERα, we identified interconnected networks of proteins with a variety of enzymatic and catalytic activities that interact not only with ERα, but also with each other. Characterization of a number of these proteins has demonstrated that, in addition to their previously identified functions, they also influence ERα activity and expression of estrogen-responsive genes.

**Conclusion:**

The agarose gel fractionation method we have developed would be useful in identifying proteins that interact with DNA-bound transcription factors and should be easily adapted for use with a variety of cultured cell lines, DNA sequences, and transcription factors.

## Background

Estrogen receptor α (ERα) is a ligand-inducible transcription factor involved in regulating expression of estrogen-responsive genes [1]. Upon binding hormone, ERα undergoes a conformational change, binds to estrogen response elements (EREs) residing in target genes, and initiates changes in gene expression. We and others have demonstrated that, in addition to the hormone-induced change in ERα conformation, the receptor undergoes another conformational change, which is induced by binding of the receptor to individual ERE sequences [2-7]. Thus, both hormone and DNA induce conformational changes in ERα structure.

ERα does not function in isolation, but serves as a nucleating factor to recruit numerous coregulatory proteins required to effectively modulate transcription. In fact, much of what we know about regulation of estrogen-responsive genes has come through the identification of ERα-associated coregulatory proteins and elucidation of mechanisms by which they influence ERα-mediated transactivation. The majority of ERα-associated coregulatory proteins have been identified through their interaction with a discrete functional domain of the receptor, most commonly the ligand binding domain (Reviewed in [8,9]. The p160 proteins steroid receptor coactivator 1 (SRC-1), transcription intermediary factor 2 (TIF-2), and amplified in breast cancer 1 (AIB1) interact with ERα in a hormone-dependent manner and enhance ERα-mediated transcription [10-17]. Both SRC-1 and AIB1 as well as CREB binding protein (CBP) and p300/CBP-associated factor (pCAF) possess intrinsic histone acetyltransferase activity that has been implicated in enhancing gene expression by modifying chromatin structure [18-24]. A large complex of proteins identified on the basis of its interaction with the thyroid hormone and vitamin D receptors has been designated as the thyroid hormone receptor associated protein (TRAP) or vitamin D receptor interacting protein (DRIP) complex [25-27]. DRIP205/TRAP 220, which anchors the DRIP/TRAP complex to nuclear receptors, interacts with ERα in a ligand-dependent manner and enhances transcription [28,29]. In addition to the numerous coactivators that enhance ERα-mediated transcription, the corepressors nuclear receptor corepressor (NCoR) and silencing mediator for RXR and TR (SMRT) bind to the antiestrogen-occupied receptor and inhibit ERα-mediated transcription by recruiting protein complexes containing Sin3 and histone deacetylases [30-34]. Thus, ERα-associated coregulatory proteins have positive and negative effects on the ability of the receptor to activate transcription.

To better understand how ERα regulates transcription of estrogen-responsive genes, we developed a novel method to isolate proteins associated with the DNA-bound receptor, which utilizes full-length ERα and endogenously-expressed nuclear proteins and takes into account DNA- and ligand-induced changes in receptor conformation. This method should be useful in isolating regulatory proteins associated with other DNA-bound transcription factors and could yield important new information about mechanisms regulating gene expression.

## Results

### Characterization of protein-ERα-ERE complexes

To isolate novel proteins that associate with ERα and might influence estrogen-responsive gene expression, we developed a method that relied on the segregation of proteins on agarose gels and was based on the capacity of these proteins to associate with the ERE-bound receptor. Using this method, we were able to take into consideration DNA-induced modulation of ERα conformation, which we have demonstrated alters recruitment of coregulatory proteins to the DNA-bound receptor [2-5]. E_2 _was also included to ensure that ligand-induced changes in receptor conformation were considered.

**Figure 1:**
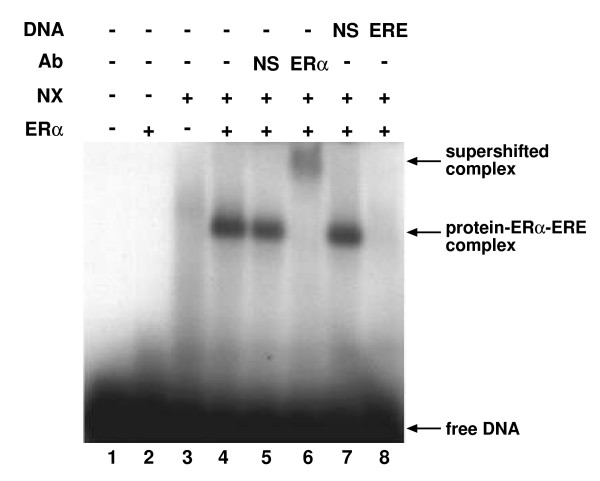
**Small-scale agarose gel electrophoresis**. ^32^P-labeled, ERE-containing oligos were incubated without (lane 1) or with ERα (lanes 2, 4–8) and/or HeLa nuclear extracts (NX, lanes 3–8). Nonspecific (NS) antibody (lane 5), ERα-specific antibody (lane 6), unlabeled oligos containing a nonspecific (NS) DNA sequence (lane 7), or unlabeled ERE-containing oligos (lane 8) were added to the binding reactions to confirm that the complexes formed were specific. 17β-estradiol (E_2_) was included in all binding reactions. Complexes were resolved on an agarose gel and visualized by autoradiography.

As seen in Fig. 1, when radiolabeled, ERE-containing oligos were fractionated on an agarose gel, neither ERα (lane 2) nor HeLa nuclear extracts (lane 3) alone produced a discrete protein-DNA complex, but when both ERα and HeLa nuclear extracts were included, a distinct, higher order protein-DNA complex was present (lane 4). The ability of an ERα-specific antibody (lane 6), but not a nonspecific antibody (lane 5), to supershift the protein-DNA complex indicated that the receptor was present in the complex and that interaction of the ERα antibody with the complex was specific. Furthermore, the ability of unlabeled ERE-containing oligos (lane 8), but not oligos containing a nonspecific DNA sequence (lane 7) to compete with the radiolabeled ERE-containing oligos confirmed the specificity of the receptor-DNA interaction. As an additional control, we utilized radiolabeled oligos that contained a nonspecific DNA sequence. While a protein-DNA complex was formed with the ERE-containing oligos, no complex was observed with the oligos containing a nonspecific DNA sequence (data not shown). Thus, this agarose-based gel fractionation method allowed us to isolate proteins that were ERα and ERE specific.

### Large scale isolation of protein-ERα-ERE complexes

**Figure 2:**
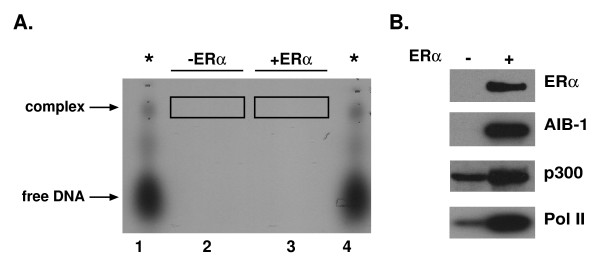
**Large-scale agarose gel electrophoresis and complex analysis**. A. Large-scale reactions containing unlabeled ERE-containing oligos were incubated with HeLa nuclear extracts and an ERα-specific antibody in the absence (lane 2) or presence (lane 3) of purified ERα. Small-scale reactions containing ^32^P-labeled ERE-containing oligos, HeLa nuclear extracts, an ERα-specific antibody, and purified ERα were also prepared and run in parallel to indicate the location of the protein-DNA complexes (*, lanes 1 and 4). E_2 _was included in all binding reactions. Complexes were resolved on an agarose gel and were visualized in the wet gel by autoradiography. Gel regions comigrating with the ^32^P-labeled protein-ERα-ERE complexes were excised (boxed areas) and contained unlabeled DNA and associated proteins without (lane 2) or with (lane 3) ERα and ERα-specific antibody. B. Proteins were isolated from the excised agarose gel pieces and subjected to Western blot analysis with antibodies directed against ERα, AIB-1, p300, or Pol II.

Once we defined the gel conditions required for formation of specific protein-ERα-ERE complexes, the next step was to increase the sample size so that sufficient amounts of protein would be available for isolation and identification. For these large-scale reactions, ERα-specific antibody was utilized to stabilize the protein-ERα-ERE complex and unlabeled oligos were used to avoid unintentional exposure of equipment to radioactive probe during the subsequent isolation and identification steps. However, small-scale samples containing radiolabeled oligos, purified ERα, HeLa nuclear extracts, and an ERα-specific antibody were run in adjacent lanes so that the position of the protein-ERα-ERE complexes in the gel could be determined (Fig. 2A, lanes 1 and 4).

Large-scale binding reactions containing unlabeled ERE-containing oligos, HeLa nuclear extracts, ERα, and an ERα-specific antibody were fractionated on one preparative-sized lane of an agarose gel and the gel region comigrating with the radiolabeled complexes was excised (lane 3, boxed area). Although distinct complexes were detected in our agarose gels when ERα, HeLa nuclear extracts, and ERα-specific antibody were included in the binding reactions (lanes 1 and 4), it seemed possible that some proteins might comigrate with the protein-ERα-ERE complex, but not actually be associated with it. Thus, a large-scale binding reaction containing the unlabeled ERE-containing oligos, HeLa nuclear extracts, and ERα-specific antibody, but no ERα, was processed in parallel and served as a negative control. The gel region comigrating with the radiolabeled protein-ERα-ERE complexes was also excised (lane 2, boxed area).

Initially, acetone or isopropanol precipitation was utilized to concentrate the proteins eluted from the agarose gel slices (data not shown). However, we found this method was unacceptable since it did not efficiently precipitate some proteins including ERα. By using a nebulizer column, which pulverizes the gel matrix and extracts the liquid and proteins, the protein recovery was far more efficient.

### Identification of known coregulatory proteins in the protein-DNA complexes

To determine whether previously identified coregulatory proteins were associated with the ERα-ERE complex or merely comigrated with it, Western analysis was carried out. As expected, ERα was detected when the purified receptor was included in the binding reaction with HeLa nuclear extracts, but not when it was omitted (Fig. 2B). AIB-1, a known p160 coactivator of ERα-mediated transcription [12], was present in the complex when ERα had been added to the binding reaction, but not when it was omitted. Although p300 and RNA polymerase II (Pol II, Refs. [35-37] were detected in the absence of the receptor, significantly more p300 and Pol II were detected when ERα had been included in the reaction. Thus, the complexes we isolated were comprised of ERα and transcription factors that are known to be involved in regulating estrogen-responsive gene expression. Furthermore, the effective association of the coregulatory proteins with the complex was dependent upon the presence of ERα.

### Identification of coregulatory proteins associated with the ERE-bound ERα

Although we had shown that previously identified coregulatory proteins were present in our protein-ERα-ERE complexes, the objective in these experiments was to identify novel proteins associated with the ERE-bound ERα. Mass spectrometry analysis was used to identify proteins present in gel regions that comigrate with the radiolabeled protein-ERα-ERE complexes (Fig. 2A, boxed areas). Numerous proteins involved in DNA replication and repair, chromatin remodeling, protein folding/stabilization, protein degradation, translation initiation and elongation, apoptosis, oxidative stress response, and signal transduction were identified (Table 1 and Additional file 1). While some proteins were identified in the absence and in the presence of ERα, significantly more peptides were recovered in the presence of ERα, as was observed in Fig. 2B with p300 and Pol II, reflecting a higher abundance of these proteins. The fact that we identified the same proteins in two or more experiments (see Additional file 1) suggests that the methods we used were reproducible. However, the most important validation of this method has come through functional characterization of these proteins. At this point, we have characterized the activity of 15 proteins associated with the DNA-bound ERα (Table 1) and found that each of these proteins influences estrogen-responsive gene expression [38-47] and unpublished data).

**Table 1:** Proteins associated with the DNA-bound ERα

**Protein**	**# of discrete peptides**	**% a.a. sequence identified**	**Effect on ERα-mediated transcription**	**References Cited**
3-methyladenine DNA glycosylase (MPG)	3	14	Decrease	[40]
apurinic endonuclease-1 (APE1)	3	18	Gene specific	Curtis and Nardulli, Submitted
flap endonuclease-1 (FEN1)	3	13	Gene specific	[42]
high mobility group protein-2 (HMG-2)	2	13	Increase	[49,50,67]
nonmetastatic protein 23 homolog 1 (NM23-H1)	5	35	Decrease	[45]
proliferating cell nuclear antigen (PCNA)	9	57	Increases basal	[43]
protein disulfide isomerase (PDI)	7	19	Gene specific	[41]
pp32	5	19	Decrease	[39]
retinoblastoma associated protein 46 (RbAp46)	6	18	Gene specific	[47]
retinoblastoma associated protein 48 (RbAp48)	6	18	Decrease	[47]
rho-GDP dissociation inhibitor α (RhoGDIα)	7	57	Gene specific	[44]
superoxide dismutase 1 (SOD1)	9	86	Increase	[46]
template activating factor 1β (TAF-Iβ)	16	38	Decrease	[38]
thioredoxin (Trx)	4	46	Gene specific	Rao and Nardulli, In Preparation
thioredoxin reductase (TrxR)	14	54	Gene specific	Rao and Nardulli, In Preparation

Selected ERα-associated proteins were characterized. The effect of each protein on ERα-mediated transcription was analyzed by transient transfection and/or RNA interference assays.

### Isolation of protein-ERα-ERE complexes using other cell lines

**Figure 3:**
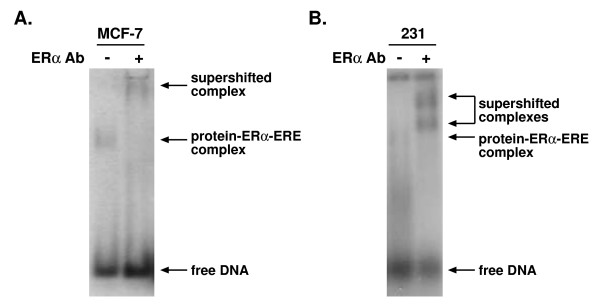
**Small-scale agarose gel electrophoresis using MCF-7 and MDA-MB-231 nuclear extracts**. ^32^P-labeled ERE-containing oligos were incubated with nuclear extracts from MCF-7 breast cancer cells, which endogenously express ERα (A), or nuclear extracts from MDA-MB-231 breast cancer cells and purified ERα (B). An ERα-specific antibody was included in the binding reaction as indicated. Complexes were resolved on agarose gels and visualized by autoradiography.

The agarose gel fractionation method we developed is not restricted in the type of cells utilized. We have used this method to form large protein-DNA complexes with nuclear extracts from MCF-7 breast cancer cells, which express endogenous ERα (Fig. 3A). Inclusion of an ERα-specific antibody supershifted the complex formed with these extracts. Interestingly, although we were unable to form a stable protein-ERα-ERE complex with purified ERα and nuclear extracts from MDA-MB-231 human breast cancer cells, which do not express ERα (Fig. 3B), inclusion of an ERα-specific antibody helped to stabilize protein-DNA complex formation. In fact, we routinely include ERα-specific antibodies to help stabilize our protein-ERα-ERE complexes.

## Discussion

We have developed a method of isolating stable protein-DNA complexes, the formation of which requires ERα, the ERE, and nuclear proteins. A number of factors were considered in establishing this methodology. First, full-length human ERα and endogenously-expressed nuclear proteins were utilized. Second, allosteric modulation of receptor conformation by DNA and hormone was taken into account by isolating proteins associated with the DNA-bound, E_2_-occupied ERα. It is, after all, the estrogen-occupied, DNA-bound receptor that recruits coregulatory proteins and initiates changes in transcription. By considering both E_2_- and DNA-induced changes in receptor conformation, we were able to identify proteins that are involved in transcriptional control and gain new insight to help define how changes in gene expression occur. Third, because traditional polyacrylamide gel shift assays do not allow large protein-DNA complexes to enter the gel [38,39,41-43,48-50], agarose gels were employed to isolate large molecular weight complexes containing ERα, ERE-containing oligos, and nuclear coregulatory proteins. In addition, low ionic strength buffer and ERα-specific antibody were used to stabilize protein-ERα-ERE complexes during the extended period of electrophoresis required. Finally, a nebulizer spin column utilized for isolating proteins from the agarose gel significantly raised the signal to noise ratio and was critical in recovering ERα and its associated proteins.

The electrophoretic agarose gel fractionation method has distinct advantages over other methods we previously used to isolate ERα-associated proteins. ERα pull-down assays were useful in identifying HeLa nuclear proteins associated with the flag-tagged ERα, but the number of proteins identified using this method was limited [38,39]. DNA affinity assays, which we used to identify a DNA glycosylase that associates with the ERE-bound ERα [40], were limited by the fact that numerous nuclear proteins bound to the ERE-containing oligos and/or agarose beads in the absence of ERα and produced a background that made it difficult to distinguish specific from nonspecific proteins. The agarose gel fractionation method allowed us to isolate a suite of ERα-associated proteins and significantly decreased the proportion of nonspecifically-bound proteins.

### Isolation of interconnected protein networks

At first glance, it might appear that much of what we have done has been to identify a number of individual proteins that interact with ERα and influence estrogen-responsive gene expression. However, one of the most fascinating findings from our agarose gel-based approach was that rather than simply identifying individual proteins that interact with ERα, we identified interconnected networks of proteins with a variety of enzymatic and catalytic activities that interact not only with ERα, but also with each other (Table 2).

**Table 2:** ERα-associated protein interactions

**Protein**	**Interacting Protein**	**Reference(s)**
APE1	ERα	Curtis and Nardulli, Submitted
	FEN1	[68]
	HMG-2	[61,69]
	NM23-H1	[61]
	PCNA	[68,70]
	pp32	[61]
	TAF-Iβ	[61]

FEN1	APE1	[68]
	ERα	[42]
	PCNA	[67,71-74]

HMG-2	APE1	[61,69]
	ERα	[49,75]
	NM23-H1	[61]
	pp32	[61]
	TAF-Iβ	[61,69]

MPG	ERα	[40]
	PCNA	[70]

NM23-H1	APE1	[61]
	ERα	[45]
	HMG-2	[61]
	pp32	[61]
	TAF-Iβ	[60]

pp32	APE1	[61]
	ERα	[39]
	HMG-2	[61]
	NM23-H1	[61]
	PCNA	[76]
	TAF-Iβ	[51-53,59,60]

PCNA	APE1	[68,70]
	ERα	[43]
	FEN1	[67,71-74]
	MPG	[70]
	pp32	[76]
	TAF-Iβ	[76]

TAF-Iβ	APE1	[61]
	ERα	[38]
	HMG-2	[61,69]
	NM23-H1	[60]
	pp32	[51-53,59,61]
	PCNA	[76]

**Figure 4:**
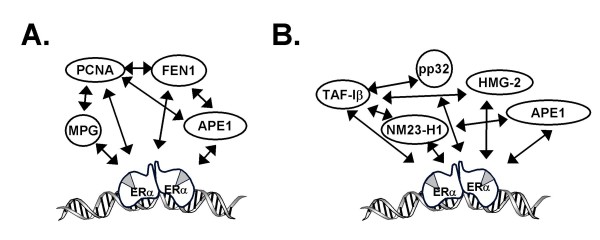
**ERα-associated proteins form interconnected networks**. A. Interactions between ERα and the DNA repair proteins MPG, PCNA, FEN1, and APE1. B. Interactions between ERα and the SET complex proteins TAF-Iβ, pp32, NM23-H1, HMG-2, and APE1.

Two examples of the ERα-associated protein networks we isolated are illustrated in Fig. 4. We identified four proteins involved in DNA repair, 3-methyladenine DNA glycosylase (MPG), apurinic endonuclease 1 (APE1), proliferating cell nuclear antigen (PCNA), and flap endonuclease, (FEN1), each of which was associated with the DNA-bound ERα and influences estrogen-responsive gene expression (Refs. [40,42,43] and C. Curtis and A. Nardulli, unpublished data) These proteins form an interactive complex of proteins (Fig. 4A and Table 2) that together are involved in base excision repair (BER). Another complex of proteins we isolated were previously identified as the SET or INHAT complex [51-53], which is comprised of template activating factor Iβ (TAF-Iβ), pp32, high mobility group protein 2 (HMG-2), APE1, and nonmetastatic protein homolog 1 (NM23-H1). These proteins form an interactive group involved in determining cell fate by initiating DNA repair or caspase-independent apoptosis (Fig. 4B and Table 2, Refs. [54,55]. Interestingly, we have characterized the effects of each of these proteins on ERα activity and found that each of these proteins influences expression of estrogen-responsive genes [38-40,42,43,45] and unpublished data).

**Figure 5:**
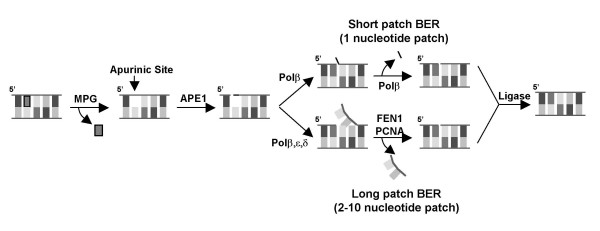
**ERα-associated proteins are involved in base excision repair**. MPG, PCNA, FEN1, and APE1 form an interconnected network of proteins involved in short- and long-patch BER.

The ERα-associated proteins we isolated are each endowed with specific activities that collectively alter basic cellular processes. As shown in Fig. 5, the DNA glycosylase MPG catalyzes the removal of a damaged or modified base and the formation of an apurinic site [56,57]. APE1 recognizes this apurinic site and initiates strand incision. DNA repair is then completed by polymerase-induced insertion of a single nucleotide and ligation. This process of replacing a single base is referred to as short patch BER. Alternatively, the DNA can be repaired through long patch BER in which FEN1 removes a short flap of nucleotides. PCNA serves as a platform for FEN1, stabilizes the interaction of FEN1 with the DNA flap, and enhances FEN1 cleavage efficiency [58]. The interaction of these DNA repair proteins with ERa is both physical and functional, but more importantly, their identification led to the discovery of an integrated protein network associated with the DNA-bound receptor that is involved in DNA repair (Figs. 4A and 5 and Table 2).

**Figure 6:**
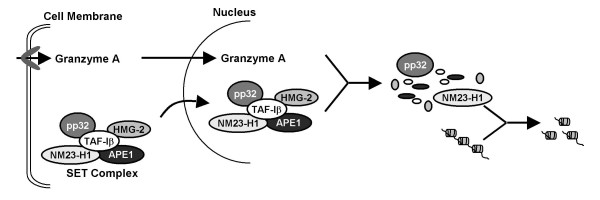
**ERα-associated proteins are in the SET complex**. TAF-Iβ, pp32, HMG-2, NM23-H1, and APE1 form an interconnected network of proteins involved in DNA repair and/or apoptosis. (Adapted from Refs. [54,55].

The SET complex is likewise comprised of an interactive group of proteins involved in regulating cellular processes, which has been described in detail by Lieberman and coworkers [52,54,59-61]. In normal cells, NM23-H1 assists in maintaining DNA integrity by nicking DNA and initiating DNA repair. In these cells, NM23-H1's DNase I activity is limited by its inhibitor, TAF-Iβ. However, a different scenario ensues when cytotoxic T lymphocytes detect a virally infected or tumor cell. In this instance, the cytotoxic T lymphocytes release Granzyme A, which enters the target cell and cleaves the inhibitor of NM23-H1, TAF-Iβ, as well as HMG-2 and APE1 (Fig. 6). With its inhibitor destroyed, NM23H1-induced DNA nicking is increased and caspase-independent apoptosis is initiated. The destruction of APE1 in these cells further hobbles the DNA repair machinery and helps to ensure that the cells undergo apoptosis. We isolated all of the SET complex proteins (pp32, TAF-Iβ, NM23-H1, HMG-2, and APE1) in our protein-DNA complexes. We and others showed previously that HMG proteins interact with ERa and other nuclear receptors, enhance receptor-DNA interaction, and increase receptor-mediated transactivation [50,62-64].

We are intrigued by the fact that the proteins we isolated interact with ERα and with each other (Fig. 4 and Table 2) providing evidence that these proteins belong to interactive networks of proteins with discrete cellular functions. The interaction of the protein networks may be fostered by the association of a protein with more than one network. For example, APE1, which interacts with ERα, components of long- and short-patch BER complexes, and SET complex proteins, may help to coordinate the actions of these protein networks and link DNA repair and transcription.

The interaction of ERα with its associated proteins may not only be physical, but may have functional consequences for both proteins. We know that MPG influences ERα-mediated transactivation and that, in turn, ERα enhances the association of MPG with modified DNA and promotes base excision [65]. Thus, by recruiting protein complexes involved in DNA repair, ERα may help to preferentially maintain the integrity of transcriptionally-active, estrogen-responsive genes.

Taken together, our findings suggest that the ERE-bound ERα serves as a nucleating factor to recruit a cohort of proteins with a variety of cellular functions that influence estrogen-responsive gene expression and that ERα may in turn enhance DNA repair and ultimately help to determine cell fate.

## Conclusion

The electrophoretic agarose fractionation protocol that we have developed provides a method to isolate interrelated networks of ERα-associated proteins involved in regulating estrogen-responsive gene expression. These studies have provided a fascinating glimpse of the complexity involved in regulating estrogen-responsive genes. This agarose gel fractionation method should be readily adaptable to a variety of cultured cell lines, DNA sequences, and transcription factors and help to define how proteins associated with DNA-bound transcription factors influence gene expression and other critical cellular processes.

## Methods

### Small scale characterization of protein-DNA complex formation

HeLa nuclear extracts and baculovirus-expressed, purified ERα were prepared as previously described [3,66]. Oligos containing the *Xenopus laevis *vitellogenin A2 estrogen response element flanked by the native DNA sequence (ERE, (5'-GAT TAA CTG TCC AAA GTC A*GG TCA *CAG *TGA CC*T GAT CAA AGT TAA TGT AA-3' and 5'-TTA CAT TAA CTT TGA TCA *GGT CA*C TG*T GAC C*TG ACT TTG GAC AGT TAA TC-3') were annealed and end labeled with ^32^γP-ATP. Radiolabeled oligos (10 pmol) were incubated with 400 fmol purified ERα in binding buffer (15 mM Tris, 0.2 mM EDTA, 80 mM KCl, 50 μM ZnCl, 5 mM MgOAc, 10% glycerol, 4 mM DTT) with 1 μg of poly dI/dC, 1 μg salmon sperm DNA, 1 μg BSA, and 10 μM 17 β-estradiol (E2) for ten minutes at room temperature. HeLa nuclear extracts (10 μg) were then added and incubated at room temperature for an additional 20 minutes. Reactions lacking ERα were run in parallel with additional BSA added to maintain constant protein concentrations. 200 ng of antibody directed against YY1 (control antibody) or ERα (sc-7341 or sc-8005, Santa Cruz Biotechnology, Santa Cruz, CA) or 10 pmol of unlabeled double-stranded oligos containing an ERE or nonspecific DNA sequence (NS, 5'-CTA GAT TAC TTC TCA TGT TAG ACA TAC TCA-3', and 5'GAT CTG AGT ATG TCT AAC ATG AGA AGT AAT CTA G-3') were included in the binding reactions as indicated. The complexes and the free DNA were separated on a horizontal 1.25% low melt agarose gel (BioRad, Hercules, CA) in a modified TBE buffer (0.45 mM Tris pH 7.9, 4.5 mM boric acid, 2 mM EDTA) containing 5 mM MgOAc at 100 volts for two hours at 4°C. The gel was dried on DE81 ion exchange cellulose acetate (Whatman, Florham Park, NJ) at 65 C for 30 minutes under vacuum and visualized by autoradiography.

### Large scale complex formation

For large scale isolation of protein-ERα-ERE complexes, DNA oligos containing the *Xenopus laevis *A2 ERE and surrounding DNA sequence were annealed and the binding reactions were incubated as described above except that they were increased to include 50 pmol DNA, 260 μg of HeLa nuclear extract with or without 18 pmol of ERα in a total volume of 200 μl. 3.2 μg of an ERα-specific antibody (sc-8002, Santa Cruz Biotechnology, Santa Cruz, CA) was added to help stabilize the ERα-containing complexes. All samples were loaded onto 10 cm × 15 cm horizontal 1.25% agarose gels prepared with molecular biology grade agarose (BioRad, Hercules, CA) and modified TBE buffer. Unlabeled ERE-containing oligos were utilized in all samples submitted for mass spectrometry analysis. Marker lanes, which contained radiolabeled oligos, 3 pmol ERα, 50 μg HeLa nuclear extract, and 0.6 μg of ERα-specific antibody in 40 μl total volumes were run in parallel at 100 V for 2 h to indicate the position of the complexes. After fractionation, the wet gel was subjected to autoradiography overnight at room temperature and the regions containing the unlabeled protein-ERα-ERE complexes were excised. Proteins were isolated with the Montage gel extraction kit (Millipore, Billerica, MA) according to manufacturer's directions. The extracted proteins were concentrated using Microcon YM-10 size exclusion columns (Millipore, Billerica, MA) with a molecular weight cutoff of 10 kDa and then subjected to mass spectrometry analysis as previously described [38]. Peptide fragments found in multiple proteins were excluded from the data analysis.

### Western analysis of ERα-associated proteins

Proteins isolated from large-scale agarose gels were fractionated on denaturing SDS-PAGE, transferred to nitrocellulose, and subjected to Western analysis. Blots were probed with antibodies specific to ERα, p300, RNA polymerase II (sc-8005, sc-585, or sc-899, respectively, Santa Cruz Biotechnologies, Santa Cruz, CA) or AIB-1 (A79920, BD Transduction Labs) and a horseradish peroxidase-conjugated secondary antibody. Proteins were visualized using a chemiluminescent detection system as previously described [4].

## Abbreviations

ERα: estrogen receptor α; ERE: estrogen response element; NCoR: nuclear receptor corepressor; SMRT: silencing mediator for RXR and TR; TIF-2: transcription intermediary factor 2; AIB1: amplified in breast cancer 1; Pol II: RNA polymerase II; MPG: 3-methyladenine DNA glycosylase; APE1: apurinic endonuclease 1; PCNA: proliferating cell nuclear antigen; FEN1: flap endonuclease 1; BER: base excision repair; TAF-Iβ: template activating factor Iβ; HMG-2: high mobility group protein 2; NM23-H1: nonmetastatic protein 23 homolog 1.

## Authors' contributions

VSL developed the initial approach of isolating protein-receptor-DNA complexes using agarose gel fractionation. The method was further refined by JRS-N and then by YSZ, who isolated the majority of the proteins identified. JRY and coworkers identified each of the receptor-associated proteins using mass spectrometry analysis and AMN guided the overall project. All authors have read and approved the final manuscript.

## Supplementary Material

Additional file 1 **ERα-interacting proteins isolated from agarose gel complexes**. HeLa nuclear extracts were incubated with ERα-specific antibody and radiolabeled ERE-containing oligos in the absence or presence of purified ERα. Isolated proteins were subjected to trypsin digestion and mass spectrometry analysis. Peptide sequences were blasted against the SEQUEST database to identify the isolated proteins. Proteins identified in more than one independent experiment are grouped by cellular function and listed by gene name, accession number, and protein description. The number of independent experiments in which each protein was identified, the number of peptides isolated, and the percent of amino acid sequence identified are indicated. Data are compiled from 4 independent experiments. Click here for file
